# Malaria Vaccine Yields Parasite-Killing Antibodies in Human Volunteers

**DOI:** 10.1371/journal.pmed.0020408

**Published:** 2005-11-08

Many adults living in malaria-endemic areas have acquired clinical immunity against the disease: even though they are constantly reinfected and frequently carry parasites, they are able to control the level of parasitemia and don't develop clinical symptoms. The passive transfer of serum IgG from such individuals can confer a similar level of protection to individuals without acquired immunity. Searching for the targets of those protective antibodies, Pierre Druilhe and colleagues had previously identified Plasmodium falciparum merozoite surface protein 3 (MSP3).

Subsequent work from Druilhe's group and others provided additional support that MSP3 might be a good candidate for a malaria vaccine: (1) naturally occurring or artificially raised antibodies against MSP3 can, in collaboration with monocytes, inhibit parasite growth in vitro, (2) an MSP3-based vaccine can confer immunity in primates (and protection correlates with anti-MSP3 antibody titers), and (3) anti-MSP3 antibody transfer can protect a malaria-susceptible humanized mouse model.

Druilhe and colleagues have now completed the first human trial of an MSP3-based vaccine. Some 30 healthy volunteers received three subcutaneous injections, each over several months of different doses of MSP3 peptide together with either aluminum hydroxide or Montanide ISA720. (Some 36 individuals were originally enrolled, but five of the recipients of MSP3 plus Montanide developed local erythema after the second injection and, per protocol specifications, received no third injection, and a sixth dropped out during the follow-up.) Details of the trial and the safety outcome will be published elsewhere (Infect Immun 73. DOI:10.1128/IAI.73.12).

Both vaccine formulations induced cellular and humoral immune responses in the majority of the volunteers, even at the lowest peptide doses. Specific T-cell responses were seen in 29 of 30 participants, vaccine-specific antibodies developed in 23 of 30, and 18 of 30 reacted with the parasite native protein. Like those of the naturally immune individuals from endemic areas, the vaccine-induced antibodies were primarily of the IgG1 and IgG3 subclasses. Antibodies elicited by MSP3 plus alum were generally of higher affinity (compared with the Montanide formulation), and similar to those from individuals with acquired immunity. Some of the MSP3 plus alum responses lasted for up to 12 months.

To test for functional immunity, Druilhe and colleagues showed that serum antibodies from those volunteers who had MSP3-specific antibodies were able to inhibit parasite growth in collaboration with monocytes. They also tested the antibody in vivo in a humanized mouse model and found that they enabled parasite clearance from the blood.

“A particular strength of the MSP3 trial is that functional assays were utilized to assess the quality of the antibody response produced,” say Brendan Crabb and James Beeson in an accompanying Perspective. However, they also point out that important questions remain, some of which will only be answered in efficacy-based trials. Encouraged by the results of the initial trial, Druilhe and colleagues have started a phase II efficacy field trial of MSP3 plus alum in malaria-exposed individuals. The results are eagerly awaited.

